# The Role of Skin and Soft-Tissue Excision in the Management of the Bulbous Nasal Tip in Asian Rhinoplasty

**DOI:** 10.1093/asjof/ojaf043

**Published:** 2025-05-22

**Authors:** Dong Cui, Zhifang Zheng, Ahmed Sharjeel, Junliang Guo

## Abstract

**Background:**

Correcting bulbous nasal tips in Asian tip plasty is challenging because of the firm and thick skin soft-tissue envelope (SSTE), and some patients may not achieve the desired results despite reshaping cartilage and bone structures.

**Objectives:**

The authors of this study evaluate the effectiveness of nasal skin and soft-tissue excision in patients with persistent bulbous tips after unsatisfactory scaffold-based rhinoplasty.

**Methods:**

From November 2019 to November 2021, the patients with bulbous nasal tips caused by firm and thick SSTE underwent nasal skin and soft-tissue excision after inadequate results from bone and cartilage scaffold reshaping. Any excess skin at the infratip lobule, the nasal columella, and the nasal ala was excised in accordance with established aesthetic proportions. The patients were followed for 12 months, with photographs taken at the final visit.

**Results:**

A total of 22 female patients were included in the current study. All patients underwent infratip lobule skin resection and interdomal sutures. Additional procedures included partial alar resection (20/22), implant-based rhinoplasty (16/22), pyriform foramen augmentation (14/22), interdomal fat pad resection (13/22), nasal bone narrowing (13/22), and partial columella excision (3/22). Three patients had reduced nasal tip projection, whereas 3 remained unchanged. Two received a cephalic trim for flared lateral crura. One year after surgery, all patients were satisfied with the results.

**Conclusions:**

Correcting bulbous nasal tips because of the firm and thick SSTE in East Asian patients may require techniques like partial nasal skin and soft-tissue resection, as reshaping cartilage and bone may be insufficient for optimal results.

**Level of Evidence: 4 (Therapeutic):**

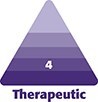

Bulbous nasal tip refers to an abnormally broad nasal tip with indistinct tip-defining points and a large space.^1^ Although bulbous nasal tips and lower dorsa are common in East Asian patients, who frequently request reshaping surgery, the correction of a bulbous nasal tip is challenging.^2^ Proper preoperative nasal evaluation and the identification of potential pitfalls are essential before formulating a surgical plan.^3^ Asians exhibit underdeveloped and thick nasal bones with a small and thin septal cartilage. Additionally, Asians have a thicker skin soft-tissue envelope (SSTE) than Caucasians, characterized by abundant subcutaneous soft tissue.^4^ Thick SSTE can obscure minor asymmetries, irregularities, and contours of cartilage grafts. However, thick skin at the nasal tip can conceal the definition achieved through cartilage manipulation and grafting because it obscures the underlying nasal framework. This can result in a rounded nasal tip that lacks refinement. Additionally, thick SSTE is associated with a higher risk of postrhinoplasty fibrosis syndrome, particularly in patients with thick sebaceous skin.^5^ Consequently, thick skin poses a significant challenge in achieving a well-defined nasal tip and may contribute to postoperative dissatisfaction.^5^ Consequently, managing bulbous nasal tips in Asians is more challenging than in Caucasians, which are commonly encountered during rhinoplasty and lead to poor cosmetic outcomes.^6,7^

In the context of rhinoplasty for East Asian patients, the surgical approach typically encompasses the establishment of a robust structural framework, contouring of the framework utilizing cartilage, and soft-tissue contouring of the nasal tip and ala.^3,8-10^ Following the reduction of the bulbous tip through suture techniques and the application of cartilage grafts, the skin at the tip undergoes a process of redraping over the cartilaginous framework. This skin subsequently experiences a gradual shrinkage and tightening over time. The phenomenon known as the “shrink-wrap” effect exhibits notable differences between thick and thin skin; specifically, thicker skin tends to conform less effectively to the underlying skeletal framework compared with thinner skin.^5^

Kim et al categorized patients with bulbous nasal tips into 3 distinct groups based on their preoperative SSTE characteristics and the dimensions of their lower lateral cartilage.^11^ Group I is defined by a bulbous morphology resulting from a relatively thin SSTE in comparison with the larger lower lateral cartilage. Patients in Group I may attain optimal outcomes through the reshaping of the cartilage and bone scaffolds. Group II is characterized by a bulbous tip appearance attributable to a thicker SSTE. This group is further subdivided based on the characteristics of the superficial musculoaponeurotic system (SMAS). Patients exhibiting a loose SMAS are classified as Group IIa, whereas those with a firm SMAS are designated as Group IIb. Flap elevation is generally more straightforward for patients in Group IIa compared with those in Group IIb. For Group IIa patients, reshaping of the cartilage framework and additional defatting procedures are essential. Conversely, in cases involving a dense SMAS, correcting the specific bulbous tip can be challenging, and postoperative complications may arise despite thorough preoperative evaluations.

The role of skin and soft-tissue resection from the infratip lobule, columella, partial alar skin resection and skin excision in the treatment of Group IIb bulbous tips in East Asian rhinoplasty continue to be a subject of debate. Some medical professionals contend that the excision of excess nasal skin can lead to a reduction in the size of nasal tips and should be commonly employed in patients exhibiting bulbous nasal tips. Conversely, others argue that excessive skin resection may result in conspicuous scarring and should be avoided. The satisfaction levels of patients with bulbous nasal tips, particularly those characterized by relatively firm and thick subcutaneous tissue, regarding the technique of excess nasal skin removal remain uncertain. This study aims to investigate the efficacy of nasal skin and soft-tissue resection in patients with Group IIb bulbous nasal tips who have not attained optimal results through the reshaping of nasal bone or cartilage scaffolds.

## METHODS

### Patients and Preoperative Evaluation

**Figure 1.**
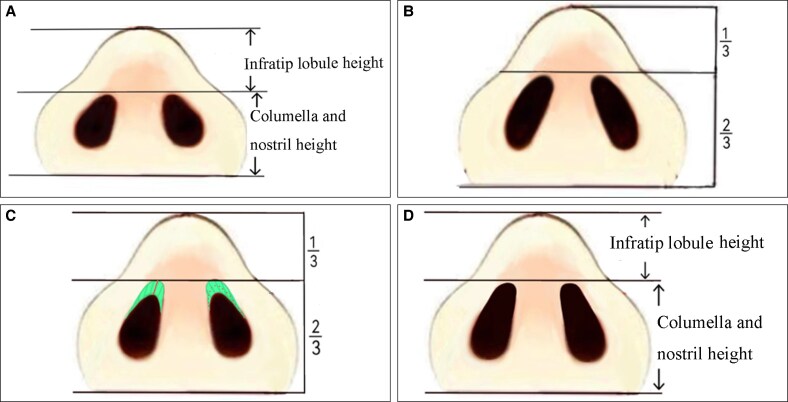
(A) In all patients, the infratip lobule height to columella and nostril height ratio of these patients exceeded the standard aesthetic ratio of 1:2. (B) The standard aesthetic ratio of the infratip lobule height: columella and nostril height ratio. (C) The reduction of excess skin in the region of the infratrip lobule used to be a treatment for bulbous nasal tips in East Asians. The red dashed line represented the length of skin excised from the nasal tip lobule, whereas the red solid line indicated the width of skin resected from the infratip lobule. (D) Crescent skin had been removed in the infratip lobule of nasal tips to achieve a standard aesthetic ratio.

**Figure 2.**
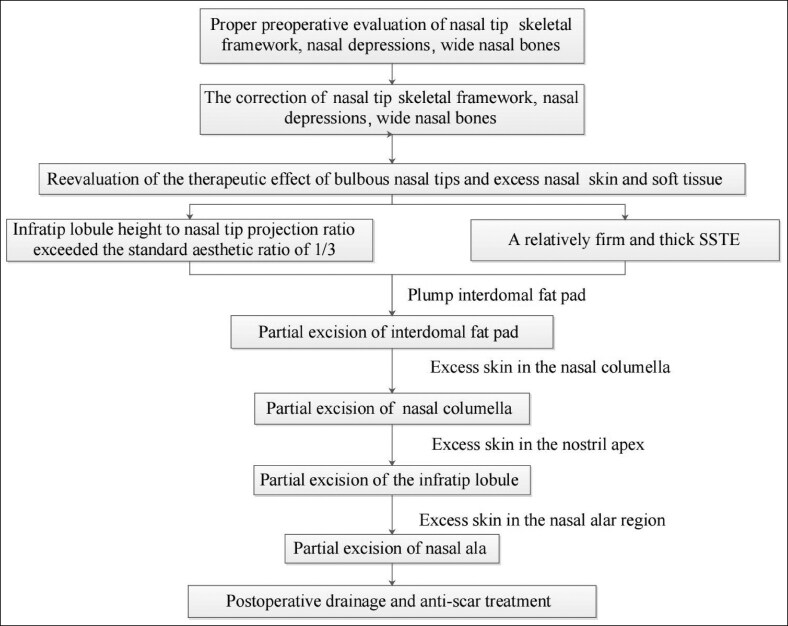
Decision tree diagram for East Asian rhinoplasty for patients with a bulbous nasal tip. If excess skin or interdomal fat was not present, then the excision step was bypassed and the next structure assessed. SSTE, skin soft-tissue envelope.

The ethics committee of the Guangzhou Hanfei Medical Beauty Hospital approved the study protocol. The inclusion criteria for this study encompassed patients presenting for rhinoplasty who exhibited bulbous nasal tips attributable to a relatively firm and thick SSTE. Specifically, the infratip lobule height to columella and nostril height ratio of these patients exceeded the standard aesthetic ratio of 1:2 (Figure 1A).^12^ A preoperative evaluation using a pinching test was conducted to assess the extensibility of the nasal tip skin and ascertain the presence of a gliding plane. If the SSTE was particularly thick, its detection could be compromised. Patients were eligible for inclusion in this study regardless of whether they had previously undergone rhinoplasty surgery.^13^ The operations were performed by the first author between November 2019 and November 2021. All patients provided informed consent for both the procedure and the study. The infratip lobule height and nasal tip projection were measured thrice using fine calipers, and the average measurement was recorded preoperatively.^14^ Various surgical techniques were selected based on the decision tree diagram (Figure 2). Patients were informed of the benefits and drawbacks associated with silicone implants and expanded polytetrafluoroethylene, enabling them to select the most appropriate prosthesis. Rib cartilage was not utilized for dorsal elevation, if the patients were unwilling to accept the longer surgical incisions and increased trauma associated with the harvesting of rib cartilage.

### Surgical Technique

#### Nasal Bone and Cartilage Reshaping

Open rhinoplasty was performed to correct nasal cosmetic deficits under general or local anesthesia. Based on aesthetic proportions and patient requirements, the nasal tips were adjusted upwards or downwards. Porous polyethylene alloplastic implants and costal cartilage grafts were used to enhance nasal tip projection. Some patients with wide nasal bones underwent simultaneous nasal osteotomy. Expanded polytetrafluoroethylene or silastic implants were used to augment the sunken nasal dorsum in the patients with saddle nose. Depressions in the pyriform foramen were filled with autologous cartilage grafts or expanded polytetrafluoroethylene biomaterials. The enlarged lateral crus of the lower lateral cartilages was partially removed. All patients had nasal interdomal sutures.

#### Skin and Soft-Tissue Removal

In this study, certain patients had previously undergone surgery and might present with significant fibrous tissue at the nasal tips. It was essential that this fibrous tissue be removed gradually. In cases where the patient has achieved the desired nasal tip height but the correction of bulbous nasal tips remains suboptimal, it was recommended that the interdomal fat pads be excised, provided that these fat pads are still present in the patients. Figure 1B illustrated the ratio of infratip lobule height to columella and nostril height, demonstrating an ideal aesthetic proportion of 1:2. The infratip lobule represented the upper one-third, whereas the columella and nostrils made up the lower two-thirds of the tip height. These aesthetic standards guided the skin removal process for the infratip lobule and the nasal columella. During the suturing of the incision on the nasal columella, it was essential to reassess the presence of excess skin in the nasal columella and the infratip lobule. Any excess skin at the apex of the nostril and the nasal columella was excised in accordance with established aesthetic proportions and the tension-free closure of wounds. Crescent-shaped excisions of skin at the new upper edge of the nostrils were marked according to the ideal infratip lobule height to nasal tip height ratio (Figure 1C), allowing for nostril shape improvement through reduced nasal tip volume (Figure 1D). Following the shaping of the nasal framework and the excision of the nasal skin in other regions of the nose, if the nasal ala appeared excessively wide and exceeded the desired aesthetic proportions (the distance between the 2 nasal alas was greater than the distance between the nasal tip and the nasal ala), it became necessary to perform a partial excision of the skin of the nasal ala. During the skin removal procedure, should any bleeding occur, it was imperative to achieve strict hemostasis through the application of electrocoagulation. Polydioxanone sutures (5-0) were used for suturing the cartilage and subcutaneous tissue, and nylon sutures (7-0) were used for closing the skin incisions. Postoperatively, the nostrils were packed with expanded sponge for 48 h, followed by nostril support devices for 3 to 4 weeks. Surgical incisions were treated with silastic gels for ∼3 months. All patients underwent nasal surgical drainage. Thicker skin was correlated with a greater volume of postoperative edema, which tended to persist for an extended duration. Consequently, surgical drainage was of paramount importance in this technique.

#### Outcome Assessment

Preoperative and postoperative measurements were conducted in triplicate by the first author under consistent conditions. Patients were followed up for 12 months. The postoperative photography and measurements were taken during their final follow-up visit. The infratip lobule height and nasal tip projection were measured postoperatively. The dimensions of both sides of the excised skin were measured and subsequently averaged for each patient. Patient satisfaction was classified as fully satisfied (++), satisfied (+), or dissatisfied (−) using a nonanonymous online questionnaire (Appendix).^15^ The customer service department of the hospital sent questionnaires to the patients through WeChat (Shenzhen Tencent Computer System Co., Ltd, China) during their final follow-up visit, and follow-up phone calls were made 1 week later to ensure questionnaire completion.

### Statistical Analysis

All the quantified data were shown as averages ± standard deviation. Statistical analyses were performed using analysis of variance with the SPSS software (ver. 21; IBM). *P* values <.05 were considered to indicate statistical significance.

## RESULTS

### Preoperative Patient Information

A total of 22 female patients were included in the current study (20-52 years old, mean age: 30.364 ± 7.371 years). Thirteen patients (59.09%) had undergone previous nasal plastic surgery (Table 1). Of the 22 patients, 3 (13.64%) complained that their nasal tips were too high, 3 (13.64%) believed their nasal tip projection and length were ideal, and 16 (72.73%) thought their nasal tips were not high enough. All participants in the study were in good health and reported no known drug allergies.

**Table 1.** The Previous Nasal Plastic Surgery

Preoperative	*n*	Proportion (%)
No prior rhinoplasty	8	36.36
Rhinoplasty through the injection of hyaluronic acid on the nasal dorsum	3	13.64
Rhinoplasty with auricular cartilage grafts and implants	8	36.36
Nasal ala reduction	1	4.55
Rhinoplasty with costal cartilage grafts and implants	2	9.09

### Surgical Process Assessment

#### Nasal Bone and Cartilage Reshaping

The nasal tip projection was reduced in 3 patients and remained unchanged in another 3 patients (Supplemental Table 1). The remaining 16 patients underwent nasal tip elevation, with rib cartilage used as a strut or septal extension graft in 15 cases and a porous polyethylene alloplastic implant in 1 case, accompanied by implant prostheses on the nasal dorsum. These findings indicated that not all rhinoplasty patients in East Asia present with insufficient nasal tip projection. In some cases, excessive tip height might require reduction procedures. All patients received interdomal sutures to correct wide intercrural angles. Three patients opted for silastic implants for the nasal dorsum, whereas the remaining patients opted for expanded polytetrafluoroethylene biomaterials. Porous polyethylene implants were covered with auricular cartilage grafts to prevent skin or mucosal penetration. Cap-shaped cartilage grafts and the muscle membrane of the rectus abdominis were used to reshape the nasal tips in 16 patients, cortical rib cartilage grafts were used in 15 patients, and an auricular cartilage graft was used in 1 patient. Two patients (9.09%) with overly flared lateral crura received a cephalic trim, with an average resection width of 3 mm (Supplemental Table 2). Fourteen patients (63.64%) had pyriform foramen depressions filled, and 13 patients (59.09%) underwent nasal bone narrowing. No implant migration from the nasal base occurred after 7 days of external fixation. All patients received negative pressure drainage to evacuate the accumulated blood.

#### Skin and Soft-Tissue Removal

In this study, some patients had previously undergone surgeries that involved the removal of their interdomal fat pads. Notably, a significant amount of proliferative fibrous tissue was observed in the subcutaneous tissue of the nasal tips in these patients, which was excised during the surgical procedure. Thirteen cases (59.09%) retained interdomal fat pads at the time of the second surgery and subsequently underwent resection of these fat pads. All patients had skin removed from the infratip lobule. Video 1 demonstrates nasal tip elevation through open rhinoplasty, followed by infratip lobule skin excision to optimize aesthetic proportions. Overall, the mean length and width of the resected skin were 8.86 ± 1.73 and 2.79 ± 1.02 mm, respectively (Supplemental Table 1). Only 3 cases (13.64%) had excess skin removal of the columella, with an average excision width of 4.66 ± 1.53 mm. Twenty patients underwent partial alar skin resection, with an average width of 3.43 ± 1.31 mm. Video 2 demonstrates the immediate postoperative outcome following: (1) nasal tip elevation, (2) osteotomy-mediated nasal bone narrowing, (3) interdomal suture placement, (4) cephalic trim of the lateral crura, (5) interdomal fat pad resection, and (6) partial resection of the excess skin at the infratip lobule, the nasal columella, and the nasal ala.

### Postoperative Effects

**Figure 3.**
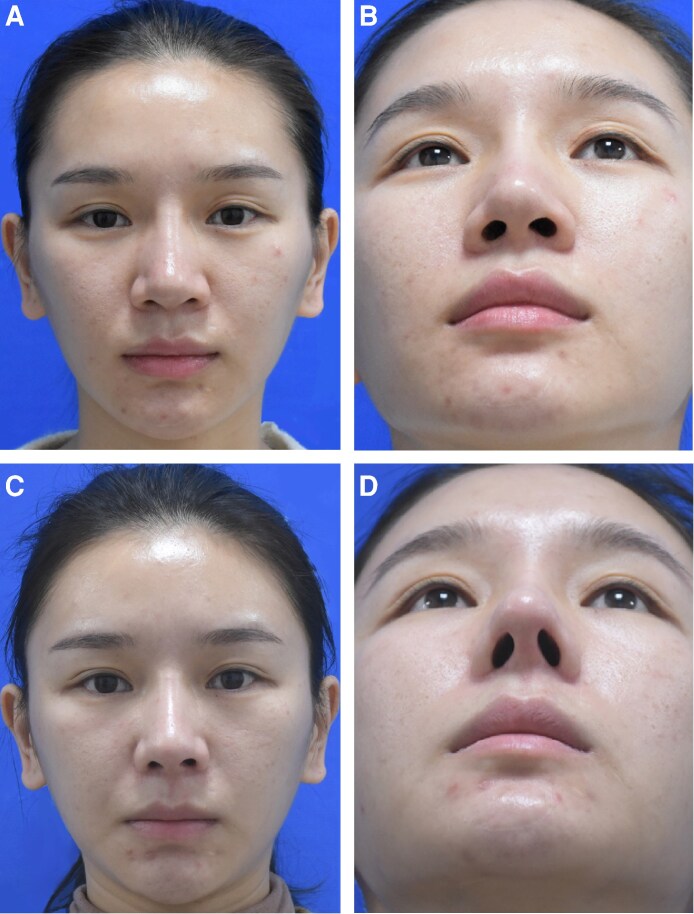
A 26-year-old woman who had a bulbous nasal tip underwent partial removal of the infratip lobule and nasal alar skin. The patient was satisfied with the treatment outcome 12 months following surgery. (A) Preoperative view, frontal. (B) Preoperative view, basal view. (C) Postoperative view at 1-year follow-up, frontal. (D) Postoperative view at 1-year follow-up, basal view.

**Figure 4.**
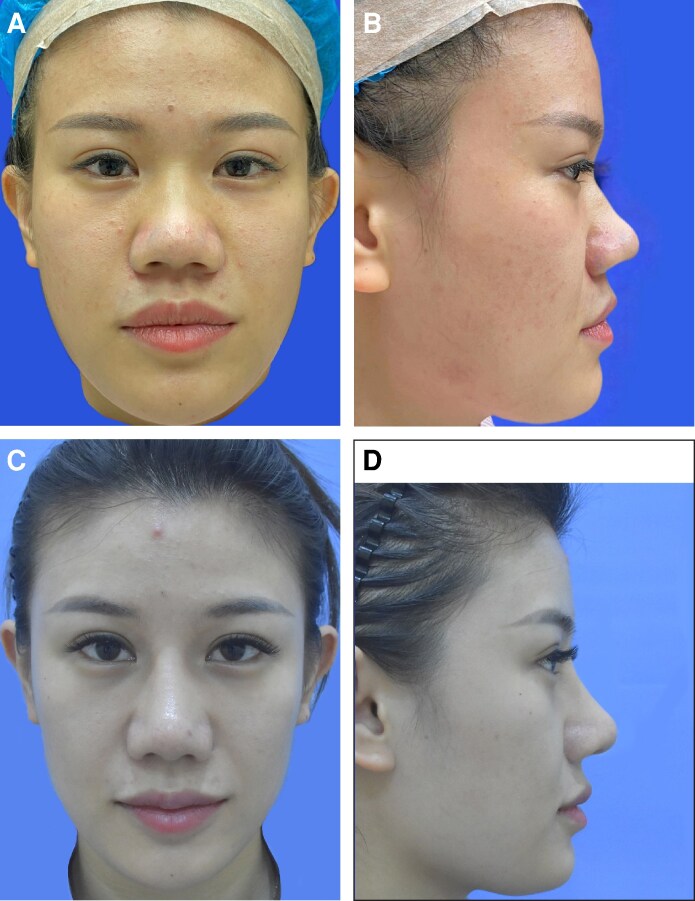
A 30-year-old woman who had a bulbous nasal tip underwent partial removal of infratip lobule skin. The patient was satisfied with the treatment outcome 1 year following surgery. (A) Preoperative view, frontal. (B) Preoperative view, left. (C) Postoperative view at 12 months follow-up, frontal. (D) Postoperative view at 12 months follow-up, left.

Nasal tip projection was reduced in 3 patients and remained unchanged in another 3 patients (Supplemental Table 2). The remaining 16 patients underwent nasal tip elevation, which significantly increased the nasal tip projection from 18.93 ± 2.89 to 24.19 ± 1.80 mm (*P* < .001). After partial removal of infratip lobule skin, there was a significant reduction in infratip lobule height, from 10.70 ± 2.34 to 7.52 ± 0.72 mm (*P* < .001). The ratio of infratip lobule height to columella and nostril height was more preoperatively (1.12 ± 0.25 mm) than postoperatively (0.46 ± 0.06 mm), approaching the aesthetic ratio of 1:2 (*P* < .001). All participants successfully completed the questionnaires. All patients were satisfied with the treatment outcomes 1 year following surgery, with no significant differences observed between the 1-year and 6-month satisfaction rates (*P* = .083). At 6 months, out of 22 patients, 3 were fully satisfied and 19 were satisfied with the results. At 1 year, 6 out of 22 patients were fully satisfied, and 16 were satisfied with the results. Preoperative and postoperative photographs of 2 patients are presented in Figures 3 and 4. All wounds healed without complications such as nasal obstruction, nasal tip deviation, or nostril deformation. Although the scars initially appeared hypertrophic, they matured and turned white within 6 months postoperatively. Three patients reported nostril asymmetry. Postoperative edema lasted for 7 to 10 days.

## DISCUSSION

The Asian nose is characterized by thick skin, abundant soft tissue, and a weak cartilage framework, resulting in underprojection of the nasal dorsum and tip.^9^ In our study, rib cartilage grafts were used in 15 cases to improve nasal tip projection, accompanied by prosthetic implants on the nasal dorsum. Only 1 case received a porous polyethylene alloplastic implant to elevate the nasal tip because of rib cartilage calcification. Cap-shaped cartilage grafts and the rectus abdominis muscle membrane were used to wrap the nasal implant's tip. When cartilage is partially ossified, surgeons face the daunting task of carving the hardened cartilage while also managing warping.^16^ Peng et al reported that porous polyethylene alloplastic implants can effectively be used to treat severe bulbous nasal tips.^17^ Cartilage flaps were first used by García-Velasco to increase the nasal tip projection.^18^ Cap-shaped grafts were obtained from cortical rib cartilage grafts, auricular cartilage grafts, or resected cartilages from the cephalic or middle portions of the lateral crura of alar cartilages.^18^

In Caucasian individuals, correction of a bulbous nasal tip typically involves the use of sutures, as well as incision or excision of the lower lateral cartilage.^19^ Lower lateral cartilage suturing techniques include transdomal sutures (to narrow individual domes), interdomal sutures, lateral crural mattress sutures (to reduce lateral crural convexity), and columella-septal sutures (to prevent tip drop and adjust tip projection).^20^ In our study, interdomal sutures were used in all patients, following Kim and Suh's method to ensure symmetry, strengthen the tip, and narrow the tip complex.^21^ However, only 2 patients with flared lateral crura of the lower lateral cartilage underwent cephalic trim, with an average resection width of 3 mm (9.09%). Gruber and Friedman suggested leaving an ∼6 mm wide lateral crus, whereas our experience indicated that retaining at least 4.5 mm of the lateral crus of the lower lateral cartilage is essential.^20^

Treating bulbous nasal tips in East Asians requires addressing not only the lower lateral cartilage, but also incorporating multiple techniques to correct voluminous thick skin, interdomal fat pads, wide and hanging alas, wide nasal bones, and retracted premaxilla. This approach aligns with Wang et al's perspective.^2^ In certain instances, partial resection of the nasal skin is essential to attain optimal outcomes, as the mere reshaping of bone and cartilage scaffolds may not produce the desired results. The authors of this study present, for the first time, the concept that the excision of multiple nasal subunit skin can serve as a beneficial adjunct to the modification of hypertrophic nasal cartilage and bone scaffolds in patients exhibiting bulbous nasal tips.

The East Asian nose is typically small with thick, voluminous skin, a low dorsum, wide and hanging ala, a bulbous tip, and a retracted premaxilla.^8^ In instances where the SMAS is characterized by thickness and density, the skin envelope exhibits inelasticity because of the substantial presence of fibrotic tissue.^11^ Consequently, the contour of the projected lower lateral cartilage is not adequately represented through subcutaneous tissue excision, often resulting in unsatisfactory postoperative outcomes. The nasal SSTE comprises several layers, including the skin, superficial fatty layer, fibromuscular layer, deep fatty layer, and perichondrium/periosteum. Notably, thick nasal skin is frequently associated with larger pores, as well as an increased thickness of both the dermis and fatty layer. However, a more significant distinction lies in the fibromuscular layer, which is considerably thicker. The excision of this fibromuscular layer constitutes a critical operative step in the management of a thick, bulbous nasal tip. Copcu et al demonstrated the surgical and radiological anatomy of the interdomal fat pad in cadavers and patients.^22^ In our study, 13 cases (59.09%) involved interdomal fat pad resection, probably because several of the patients had already undergone interdomal fat pad removal preoperatively. Mohebbi et al also reported that this procedure could lead to higher postoperative satisfaction with the aesthetic outcome compared with rhinoplasty alone in patients with moderate or thick nasal tip skin.^23^ The dermal component of dense tip skin should not be forcibly removed, because this may damage the subdermal plexus, which is primarily responsible for the blood supply to the nasal tip, potentially leading to vascular complications.^13^

It is seldom reported that the reduction of the skin of the infratip lobule, nasal columella, and nasal ala can effectively treat bulbous nasal tips. In our study, all patients presenting with bulbous nasal tips underwent reshaping of the cartilage and bone scaffolds; however, the desired outcomes were not achieved. The decision to excise a portion of the nasal columella skin should be contingent upon the suture tension associated with the incision. In the case of a leptorrhine nose, the basal view is characterized by an isosceles triangle, where the lobule constitutes the upper third of the triangle, whereas the columella and nostrils comprise the lower two-thirds. In accordance with this aesthetic ratio and the tension of the skin incision suture, we executed a reduction of the skin in the infratip lobule and nasal columella. The excision of the nasal ala skin typically represents the final step in the rhinoplasty procedure. It is advisable to refrain from removing any portion of the nasal ala skin unless absolutely necessary, as such removal may compromise the blood supply to the nasal tip. This aspect of our approach is particularly noteworthy, because it is not widely accepted in current practice. It is important to note that the reduction of the skin in the infratip lobule was deemed unsuitable for patients exhibiting alar rim collapse. The long axis of the nostrils was elongated, aligning with the aesthetic proportions reported by Ellenbogen and Bazell.^24^ It is believed that the midpoint of the infratip lobule should coincide with the nostril apex, dividing the tip-defining points and the columellar–lobular angle, consistent with our findings.^25^ In our study, the ratio of infratip lobule height to columella and nostril height increased postoperatively, approaching the aesthetic ratio of 1:2 (*P* < .001). Preserving the infratip lobule tissue lining around the nostrils is essential to maintain normal nostril morphology. The asymmetry of the nostrils is a relatively common occurrence. Three patients have reported experiencing nostril asymmetry. McKinney and Stalnecker suggested that a portion of the arch should be resected without removing the lining if there is a bulbous appearance because of a flared arch but the skin is thick and the nasal projection is adequate.^26^ Although excising the entire skin, it is important to keep the incision arcuate rather than straight to prevent scar contracture. Only 3 cases (13.64%) had excess skin removal of the columella, with an average excision width of 4.66 mm. Some Asians with platyrrhine or mesorrhine noses could also have excess columella skin.

In our study, 20 of the 22 patients underwent partial nasal ala resection, highlighting its necessity for severe bulbous nasal tips. One of the most significant challenges surgeons face is the unpredictability of nasal alar skin reduction outcomes. Surgeons must carefully balance the risk of scar formation against the potential for minor improvements. The use of fine intradermal suturing techniques is essential to minimize scar formation on the nasal ala. Nostril support devices for 3 to 4 weeks after surgery are necessary to maintain the nostril shape.

This study had several limitations. First, the relatively small sample size limited statistical power, necessitating larger scale investigations. Although no long-term complications were observed with silastic implants, porous polyethylene, or expanded polytetrafluoroethylene during the 12-month follow-up, this duration was potentially insufficient for comprehensive risk assessment. Second, the findings were primarily applicable to East Asian populations, and their generalizability to Caucasian patients required further validation. Third, the inclusion of both primary and secondary rhinoplasty cases introduced confounding variables because of differences in surgical complexity. Finally, nonstandardized techniques prevented definitive attribution of outcomes to specific maneuvers.

## CONCLUSIONS

For East Asian patients, bulbous nasal tip reshaping is a challenging aspect of rhinoplasty that involves the correction of voluminous thick skin, a low dorsum, wide and hanging alas, wide nasal bones, flared lateral crura, and a retracted premaxilla. In certain instances, partial resection of the nasal skin and soft tissue may be required to attain optimal outcomes, because the mere reshaping of bone and cartilage scaffolds may not produce the desired results. Our study found that the recovery process was rapid, complications were minimal, and satisfaction rates were high in this treatment.

## Supplementary Material

ojaf043_Supplementary_Data
