# Supplementary material for: The Role of Skin and Soft-Tissue Excision in the Management of the Bulbous Nasal Tip in Asian Rhinoplasty
Source: Aesthet Surg J Open Forum. 2025 May 22;7:ojaf043. doi: 10.1093/asjof/ojaf043 (PMC12378440; doi:10.1093/asjof/ojaf043)
Supplement: ojaf043_Supplementary_Data [file ojaf043_Supplementary_Data.zip › Supplementary_table_1.doc]

**Supplementary table 1**. Quantitative evaluation after surgery

| No. | Height of the infratip lobule preoperatively (mm) | Height of the infratip lobule postoperatively(mm) | Height from the nasal tip to nasal columella base preoperatively (mm) | Height from the nasal tip to nasal columella base postoperatively(mm) | Ratio of infratip lobule height to columella and nostril height preoperatively | Ratio of infratip lobule height to columella and nostril height postoperatively | Patient Satisfaction 6 months after surgery | Patient Satisfaction one year after surgery |
| --- | --- | --- | --- | --- | --- | --- | --- | --- |
| 1 | 10.0 | 7.5 | 23.0 | 23.0 | 0.77 | 0.49 | + | + |
| 2 | 11.0 | 7.0 | 21.0 | 23.0 | 1.10 | 0.44 | ++ | ++ |
| 3 | 7.5 | 7.0 | 15.0 | 24.0 | 1.00 | 0.41 | + | + |
| 4 | 8.0 | 6.0 | 14.0 | 22.0 | 1.33 | 0.38 | + | + |
| 5 | 10.0 | 7.0 | 18.0 | 23.0 | 1.25 | 0.44 | + | ++ |
| 6 | 16.0 | 8.0 | 27.0 | 24.0 | 1.46 | 0.50 | + | ++ |
| 7 | 15.0 | 8.0 | 25.0 | 30.0 | 1.50 | 0.36 | + | + |
| 8 | 10.0 | 7.0 | 18.0 | 25.0 | 1.25 | 0.39 | + | + |
| 9 | 11.0 | 7.5 | 19.0 | 24.0 | 1.38 | 0.46 | + | + |
| 10 | 14.0 | 9.0 | 25.0 | 23.0 | 1.27 | 0.64 | ++ | ++ |
| 11 | 15.0 | 8.0 | 25.0 | 25.0 | 1.50 | 0.47 | + | + |
| 12 | 12.0 | 7.5 | 24.0 | 24.0 | 1.00 | 0.46 | + | + |
| 13 | 10.0 | 7.0 | 20.0 | 23.0 | 1.00 | 0.44 | + | ++ |
| 14 | 11.0 | 9.0 | 18.0 | 25.0 | 1.57 | 0.56 | + | + |
| 15 | 9.0 | 8.0 | 17.0 | 25.0 | 1.13 | 0.47 | + | + |
| 16 | 10.0 | 7.0 | 23.0 | 24.0 | 0.77 | 0.41 | + | + |
| 17 | 8.0 | 7.0 | 16.0 | 23.0 | 1.00 | 0.44 | + | + |
| 18 | 10.0 | 8.0 | 22.0 | 25.0 | 0.83 | 0.47 | + | + |
| 19 | 9.0 | 7.0 | 18.0 | 24.0 | 1.00 | 0.41 | + | + |
| 20 | 10.0 | 7.0 | 24.0 | 20.0 | 0.71 | 0.54 | ++ | ++ |
| 21 | 9.0 | 8.0 | 19.0 | 23.0 | 0.90 | 0.53 | + | + |
| 22 | 10.0 | 8.0 | 20.0 | 24.0 | 1.00 | 0.50 | + | + |
