# Supplementary material for: The Role of Skin and Soft-Tissue Excision in the Management of the Bulbous Nasal Tip in Asian Rhinoplasty
Source: Aesthet Surg J Open Forum. 2025 May 22;7:ojaf043. doi: 10.1093/asjof/ojaf043 (PMC12378440; doi:10.1093/asjof/ojaf043)
Supplement: ojaf043_Supplementary_Data [file ojaf043_Supplementary_Data.zip › Supplementary_table_2.doc]

**Supplementary table 2. Quantitative evaluation of skin or cartilage removal during the surgical process**

| No. | Age (year old) | Width of skin resected from the infratip lobule (mm) | Length of skin resected from the infratip lobule (mm) | Width of skin resected from nasal ala (mm) | Width of skin resected from nasal columella (mm) | Width of cartilage resected from lateral crus of the lower lateral cartilage (mm) |
| --- | --- | --- | --- | --- | --- | --- |
| 1 | 25 | 3.0 | 10.0 | 3.0 | 0 | 0 |
| 2 | 32 | 3.0 | 8.0 | 2.0 | 0 | 0 |
| 3 | 40 | 2.0 | 8.0 | 3.0 | 0 | 0 |
| 4 | 33 | 2.0 | 8.0 | 3.0 | 0 | 0 |
| 5 | 32 | 3.0 | 10.0 | 0 | 0 | 0 |
| 6 | 33 | 2.0 | 10.0 | 1.5 | 0 | 0 |
| 7 | 52 | 3.0 | 10.0 | 8.0 | 0 | 0 |
| 8 | 23 | 2.5 | 10.0 | 5.0 | 0 | 0 |
| 9 | 28 | 3.0 | 10.0 | 5.0 | 5.0 | 3.0 |
| 10 | 28 | 2.0 | 8.0 | 2.0 | 6.0 | 0 |
| 11 | 26 | 6.0 | 11.0 | 5.0 | 0 | 0 |
| 12 | 30 | 4.0 | 8.0 | 5.0 | 0 | 3.0 |
| 13 | 20 | 4.0 | 10.0 | 8.0 | 0 | 0 |
| 14 | 30 | 1.5 | 7.0 | 2.0 | 0 | 0 |
| 15 | 41 | 4.0 | 12.0 | 6.0 | 0 | 0 |
| 16 | 23 | 3.0 | 10.0 | 3.0 | 3.0 | 0 |
| 17 | 25 | 2.0 | 4.0 | 3.0 | 0 | 0 |
| 18 | 30 | 2.0 | 8.0 | 0 | 0 | 0 |
| 19 | 32 | 2.0 | 7.0 | 3.0 | 0 | 0 |
| 20 | 20 | 3.0 | 10.0 | 2.0 | 0 | 0 |
| 21 | 29 | 2.0 | 8.0 | 3.0 | 0 | 0 |
| 22 | 36 | 2.5 | 8.0 | 3.0 | 0 | 0 |
